# High phenotypic and phytochemical diversity of *Bactris gasipaes* (Arecaceae) fruits in Ecuador

**DOI:** 10.1371/journal.pone.0342904

**Published:** 2026-03-26

**Authors:** David Romero-Estévez, Thomas L.P. Couvreur, Michael Ayala, Eduardo Valarezo, Jorge G. Figueroa, María Judith Terán, Álvaro Rivera, Rommel Montúfar

**Affiliations:** 1 Centro de Estudios Aplicados en Química (CESAQ), Pontificia Universidad Católica del Ecuador (PUCE), Quito, Pichincha, Ecuador; 2 DIADE, Université de Montpellier, CIRAD, IRD, Montpellier, Occitanie, France; 3 Facultad de Ciencias Exactas, Naturales y Ambientales, Pontificia Universidad Católica del Ecuador (PUCE), Quito, Pichincha, Ecuador; 4 Facultad de Ciencias Agrícolas, Universidad Central del Ecuador (UCE), Quito, Pichincha, Ecuador; 5 Departamento de Química, Universidad Técnica Particular de Loja, Loja, Ecuador; 6 Laboratorio de Fitopatología, Escuela Superior Politécnica del Chimborazo (ESPOCH), Riobamba, Chimborazo, Ecuador; Institute for Biological Research, University of Belgrade, SERBIA

## Abstract

Climate change has negatively impacted food availability and nutritional quality. To address this challenge, it is essential to emphasize species that, despite their longstanding use by indigenous communities, remain underutilized or neglected. Integrating these culturally domesticated species into local food systems offers a promising strategy to improve access to healthy and sustainable foods. One such species is *Bactris gasipaes* Kunth, whose domesticated variety holds significant but largely untapped nutritional potential. Nevertheless, substantial gaps persist in our understanding of the morphological and phytochemical characteristics of its fruits. In the present study, 110 fruit samples of different *Bactris gasipaes* var. *gasipaes* individuals were analyzed to assess the proximal and biochemical composition of the mesocarp. Particular attention was given to the relationship between compositional traits and phenotypic characteristics, especially exocarp color. The results showed substantial intraspecific variation in fruit morphology and coloration, alongside notable nutritional values: crude protein (3.0–10.2%), crude fiber (0.2–3.0%), total oil (3.4–27.3%), tocopherols (<2.1 mg/g), β-carotene (<27.3 mg/100 g), and polyunsaturated fatty acids (4.7–44.1%). These results highlight the nutritional potential of *B. gasipaes* and its relevance for diversifying healthy food sources. Furthermore, results showed noticeable phytochemical differences and no strong correlations between specific components and phenotypic traits among fruit types in proximal parameters, nutritional content, and primary exocarp color, reinforcing the species’ value for food system integration. The observed diversity underscores the nutritional relevance of *B. gasipaes* from Ecuador and highlights the need for further research to classify fruit diversity for targeted use in food systems.

## Introduction

Climate change undermines food security and the quality of diets, causing multiple forms of malnutrition [1]. The food systems in rural areas of the Neotropics rely on native, introduced, or domesticated plant species, providing both tangible and intangible benefits to local communities. These species and their derivatives are associated with low-input practices and ecological knowledge [2], often fostering cultural relationships with local communities and contributing to the sustainable management of the landscape [2]. However, most of these species remain neglected or understudied in the tropics [3,4]. The loss of this agrobiodiversity involves the erosion of traditional management practices, the domestication or adaptation of intraspecific biodiversity, and the loss of social bonds and the landscape's history.

The Neotropics are recognized as a major region for Arecaceae family diversification, with over 800 recorded species [5] with a variety of associated uses [6]. Despite this diversity, only one species has been fully domesticated: *Bactris gasipaes* Kunth [7,8]. Botanical studies [9–11] have identified two varieties of this species: *B. gasipaes* var. *chichagui* (H. Karst.) A.J. Hend., denoting the wild type [12], and *B. gasipaes* var. *gasipaes*, representing the domesticated form and widely cultivated throughout the lowland neotropics (hereinafter *gasipaes,* also named “peach palm”). The primary domestication syndrome is associated with fruit characteristics, with *var. gasipaes* exhibiting larger and more fleshy fruits than var. *chichagui*; however, this may not be the only difference [11–13]. Both *gasipaes* and *chichagui* contribute to local food systems in many rural Neotropical areas [14].

**Fig 1:**
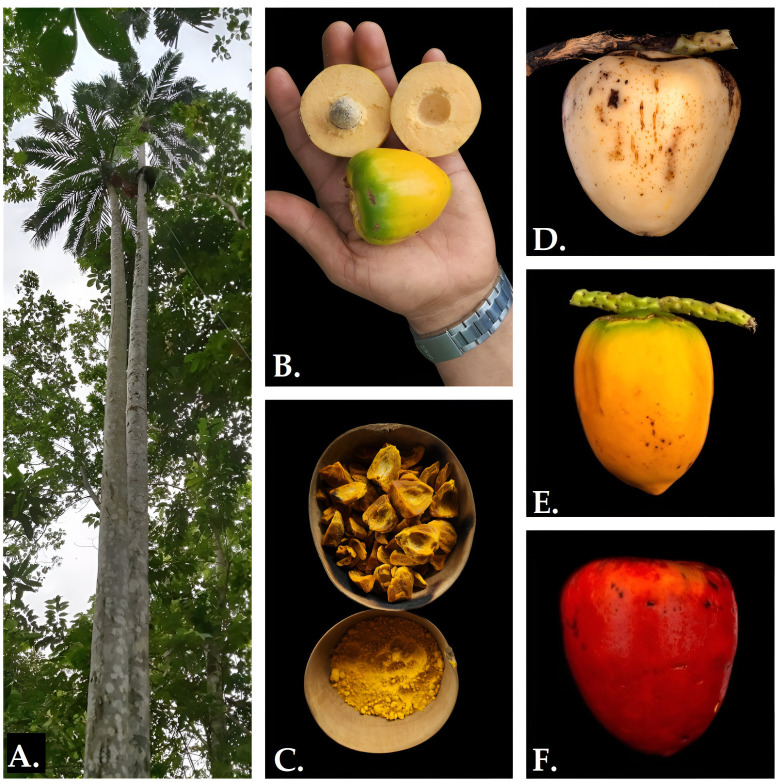
A) *Bactris gasipaes* var. *gasipaes* individual from Napo province. B) exposed mesocarp of var. *gasipaes* fruit; C) cooked and dried var. *gasipaes* fruits and flour for local consumption; D) white, E) yellow, and F) red var. *gasipaes* fruits.

*B. gasipaes* palm is an arborescent (until 25 m), allogamous, and cespitousous palm species widely distributed in the tropics. Their fruits display a high variability in shape (i.e., spherical, pyramidal, or ovoid), size, weight, and exocarp colors, e.g., red, yellow, green, orange, white, dark purple, and mixed; [15] (Fig 1). The trunk has segments intercalated by spines; some individuals present trunks without spines. Gasipaes variety is highly dependent on anthropogenic activities and is one of the major crops in small and ancestral agroforestry systems (2000–40000 m^2^), locally known as *chakras* in Ecuador [16].

Comparative analyses of lipids and other components present in *gasipaes* fruits are scarce in the scientific literature. Santos et al. [17] described biochemical differences among fruit colors (red, yellow, green, and white) from samples purchased at a local market in Belém (Brazil) to support their use in the production of functional and nutraceutical foods. Meanwhile, Carvalho et al. [18] found that red fruits from Manaus (Brazil) were preferred for consumption because of their rich unsaturated fatty acids (UFA, which enhance the absorption of provitamins), carotenoids (essential precursors of vitamin A), and tocopherols (members of the vitamin E family) content. Dias Soares et al. [19] showed that white-colored fruits purchased at a market from Belém (Brazil) are a significant source of calcium, magnesium, and phosphorus, although they have a lower carotenoid content than more vividly colored fruits. Other studies investigating biological diversity have relied on germplasm collections [13,20,21] without discriminating the fruits by their primary exocarp color.

Understanding the variability of fruits with different exocarp colors is crucial for bioeconomic ventures involving *gasipaes* fruit in the region, and the foundation of any transformation process of the natural resource lies in its biochemical characteristics [22]. For instance, *gasipaes* could be regarded as an oil crop given its high 7% dry-weight oil content [21,23]; likewise, it represents a compelling economic and nutritional alternative compared to many other locally consumed oil sources (e.g., *Elaeis guineensis)* [24]. Additionally, it is an important source of high-quality starch [25] and other minor nutrients for the local diets [17]. This biochemical diversity associated with exocarp color and its spatial scale (from agrosystems to the regional level) has been poorly explored. Previous research has examined the industrial potential of *Bactris gasipaes* variability [22,24,26–29]; however, studies addressing the biochemical composition of the mesocarp in different fruit types remain scarce in the literature.

Notably, the Ecuadorian Amazon harbors a high phenotypic diversity of *gasipaes* fruits [12,13]. This diversity is associated with chakra systems and other anthropogenic spaces (e.g., small- and large-scale commercial plantations). In some cases, up to 12 distinct fruit types can be found within a single chakra area (Montúfar, pers. obs.), indicating intense selective pressure favoring phenotypic diversity in small-scale cultivation systems. Not only is the exocarp color the primary driver of diversity, but also the presence of spines on the trunk, the capacity to grow at different altitudes (from 500 to 1200 m above sea level), and other ecological adaptations. In this region, *gasipaes* is a multi-purpose palm tree that provides food (fruits and palm hearts), construction materials (trunk, spines, leaves), and substrate for larvae (*chontacuros*), and also plays an essential role in the local culture [30].

This study aimed to explore the phenotypic and phytochemical variability of *Bactris gasipaes* var. *gasipaes* sourced from small-scale agroforestry systems (*chakras*) in the northern Ecuadorian Amazon. Our specific objectives were i) to characterize the phenotypic variability of individuals and fruits; ii) to determine the proximal parameters―dry matter, crude protein, crude fiber, total oil, and minerals―and the content of phytochemical compounds―β-carotenoid, total tocopherol, fatty acid (FA), and sterol profiles―present in the mesocarp; and iii) to explore the association between these phytochemical properties and fruit exocarp color.

## Materials and methods

### Sample collection and preparation

To explore the phytochemical and phenotypic diversity, samples representing the most prominent fruit types available in the region were analyzed. A total of 110 infructescences were collected, each from a different *Bactris gasipaes* var. *gasipaes* individual (i.e., representing 110 independent progenies), sampled across the northern Ecuadorian Amazon, including the provinces of Napo, Sucumbíos, Pastaza, and Orellana. Of these, 79 infructescences were obtained directly from local agroforestry systems (*chakras*) within the study area, and the remaining 31 infructescences were purchased from indigenous markets in Lago Agrio and El Coca. Each infructescence belonged to a distinct individual (see S2 Table for phenotypic characteristics). The phenotypical features for the 31 market individuals were unknown. All market samples originated from nearby local chakras.

Harvesting was carried out with the assistance of local farmers, who provided expertise in determining the appropriate maturation stage of each infructescence.

For phenotypic trait characterization and subsequent laboratory analyses, 110 composite samples consisting of 10–30 fruits (depending on size) from each infructescence were used.

This project was conducted in full compliance with Ecuadorian legal regulations, within the framework of the agreement on access to genetic resources established by the Government of Ecuador, in coordination with the Ministry of the Environment (MAE), under reference MAE-DNB-CM-2018–0082.

### Phenotypic variables determination

Morphological variables were recorded only for chakra-collected samples (79 samples); such data were not obtainable for market samples (31 samples), this data includes trunk height (m; measured from the base to the point where the leaves begin), number of trunks, circumference at breast height (cm), presence/absence of spines on the trunk, number of infructescences, and number/estimated number of fruits per infructescence. In most cases, the number of fruits per infructescence was directly counted; in some instances, it was estimated. We gathered 10–30 fruits (depending on size) from each infructescence, aiming to cover the range of sizes observed. We verified the selected fruits did not show signs of infection, pest damage, or advanced maturation (e.g., soft texture or visible exocarp oxidation). Immediately following collection, fruit characteristics were determined in 5–10 fruits, including average weight (g), average length (cm), average diameter (cm), and average volume (cm³). To define the exocarp color, fruits were categorized into major color groups: green, yellow, red, orange, dark purple, and white. In many cases, fruits exhibited multiple colors; in such instances, the primary (the most prominent) and secondary colors were identified.

### Proximal and chemical analyses

For proximal and chemical analyses, methodologies are described in the S1 Appendix [31–40]. The selected fruits were washed, disinfected with ethyl alcohol, and stored at 4 °C in the dark until they arrived at the Centro de Estudios Aplicados en Química of the Pontificia Universidad Católica del Ecuador (CESAQ-PUCE) in Quito. Subsequently, a representative amount of the mesocarp was extracted from several random fruits, cut into small pieces, and frozen at −20 °C for freeze-drying in a freeze-drying system (Labogene, Lillerød, Denmark) and milling (Tube Mill control, IKA, Staufen, Germany; 120 seconds at 10,000 rpms). For the proximal analysis, the cut mesocarp samples were crushed (particle size < 5 mm) and dried in a forced-air oven (Binder FD 115, Tuttlingen, Germany) according to the AOAC 934.01 reference method [41]. The analyses of ash content, crude protein, crude fiber, and minerals (S1 Appendix) were carried out in the laboratories at the Universidad Central del Ecuador. The determination of total lipids, tocopherols, FAs, and sterols (S1 Appendix) was conducted at CESAQ-PUCE. Lastly, the analysis of β-carotene content (S1 Appendix) was carried out at the Universidad Técnica Particular de Loja.

### Statistical analyses

Means and standard deviations were calculated for the replicate samples for all measured parameters. A Kruskal-Wallis test was applied to evaluate significant differences among independent samples for each color, excluding purple (n = 2) and white (n = 1) due to the limited number of available samples. Given the data distribution and after assessing parametric alternatives, Spearman’s nonparametric correlation was used to examine the relationships between phenotypic and phytochemical parameters of the samples (n = 110). A rectangular matrix with 110 fruits and 37 phenotypic and biochemical variables was constructed for analysis by Principal Component Analysis (PCA) using Euclidean distances. Phenotypic, proximal, and biochemical variables were standardized and later transformed using the Z-score normalization method. All statistical analyses were performed with SPSS (version 28.0, IBM, Armonk, USA) and Python 25.2.240.0, (Python Software Foundation, Fredericksburg, USA). No data points from the analyzed parameters were eliminated as outliers because each corresponded to a different individual; thus, they were assumed to contribute to the observed variability.

## Results and discussion

### High phenotypic variability within *gasipaes* populations

**Fig 2:**
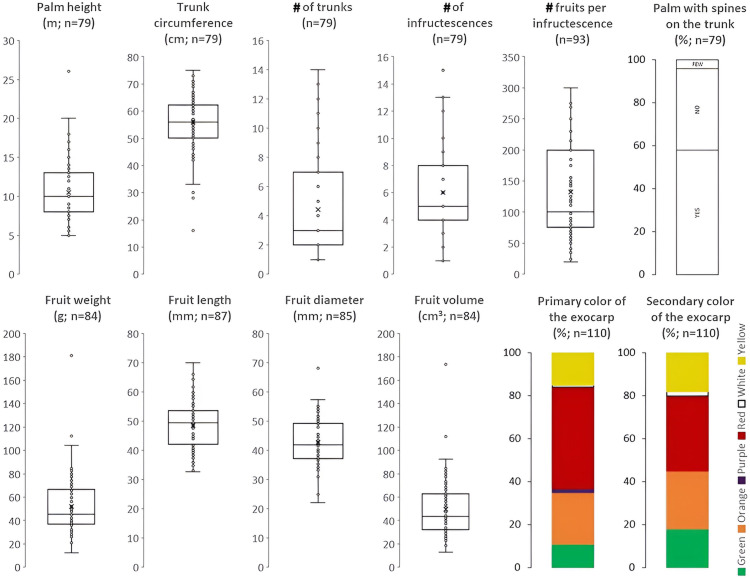
Phenotypic variation of *B. gasipaes* var. *gasipaes* individuals.

*Gasipaes* samples exhibited high phenotypic variability (Fig 2, S2 Table), consistent with findings from Colombian and Brazilian populations [42,43]. The variability, expressed as the coefficient of variation (CV), was relatively high for most of the morphological characteristics analyzed. Notably, the number of trunks per individual showed considerable variation, averaging 4 ± 3 trunks (CV = 76.2%). In addition, the number of fruits per infructescence varied significantly (20 − 300, CV = 54.3%), indicating high variability in productivity (S2 Table). These morphological differences may be attributed not only to historically induced domestication but also to the diverse abiotic conditions in the study area (e.g., edapho-climatic conditions, soil nutrient availability, altitude), growth stage, and management practices [44].

In particular, the diversity of exocarp colors [red, yellow, orange, white, green, and intermediate colors, including dark purple (S3 Fig)], exocarp patterns (with stripes, veins, or some ornament), and shapes (conical, oval, or spherical) reflects its role as the primary trait of the domestication syndrome. Moreover, considerable variability is observed even within each color category, as individual fruits frequently exhibit additional hues or intricate patterns beyond their main classification, resulting in a complex and heterogeneous appearance at the fruit level. Similarly, the presence or absence of spines constitutes a conspicuous morphological characteristic related to selection processes [45]. Spineless individuals suggest multiple independent domestication developments [21]. In the study area, nearly half of the sampled individuals had spines in their trunks (n = 49). In their ancestral state, species of the *Bactris* genus (e.g., the subtribe Bactridineae) [46] typically exhibit dense spine coverage. The reduction or elimination of spine marks is a crucial selection stage, facilitating fruit harvesting, reducing injuries, and enabling better integration into agroforestry systems [21]. However, the loss of spines may intensify the predatory actions of large insects or small mammals, impeding water evacuation from the stem and thereby facilitating the growth of epiphytic and parasitic plants.

The fruits’ phenotypic attributes have influenced their final use, even shaping demand in local markets. For example, the striped fruit (with veins) is preferred in Costa Rica, although variations in morphology do not necessarily indicate lower quality [24]. In our study area, for instance, red-colored fruits are particularly sought after, chiefly for their use in *preparing chicha* (an alcoholic beverage derived from the fermentation of carbohydrates).

The colors of both the exocarp and the mesocarp are closely associated with the presence of various phytochemical compounds, principally chlorophyll and carotenoids within chloroplasts and chromoplasts [47]. Typically, the final hue results from complex combinations and overlays of all possible substances, including waxes and lipids on the surface, which contribute to coloring [47,48].

### Intraspecific variability of proximal determination of mesocarp from *gasipaes* fruits

Mesocarp samples presented a wide range of values for moisture content (38.0–74.8%), ash (0.8–2.7%), crude protein (3.0–10.2%), total fiber (0.2–3.0%), and oil content (3.4–27.3%, Table 1) were similar to those found by previous studies [14,23,24,49–51]. Although the crude protein content was low (4.9 ± 1.02), previous research has indicated that it contains all essential amino acids required for human nutrition [29,44,52]. The samples also contained higher levels of indigestible fiber, making them attractive for consumption, as their fermentation by intestinal microorganisms produces short-chain fatty acids (acetate, propionate, and butyrate), which help regulate gut function, enhance nutrient absorption, and even influence systemic health [53–55].

**Table 1:** Results of the proximal composition analysis of mesocarp from gasipaes fruits (n = 110), including mean, standard deviation, and range of values, expressed as percentage of dry matter basis.

Parameters (%)	Mesocarp primary color						
	Green (n = 11)	Orange (n = 24)	Purple (n = 2)	Red (n = 47)	White (n = 1)	Yellow (n = 25)	All samples (n = 110)
Moisture content	55.0 ± 5.85 46.9 − 66.2	53.1 ± 7.64 38.0 − 70.6	47.1 ± 9.54 40.3 − 53.8	54.4 ± 5.21 43.0 − 74.1	74.8 --	54.2 ± 6.86 42.6 − 68.0	54.2 ± 6.58 38.0 − 74.8
Ash content	1.4 ± 0.30 0.9 − 1.9	1.5 ± 0.47 0.8 − 2.7	1.3 ± 0.43 1.0 − 1.6	1.5 ± 0.31 0.9 − 2.4	2.4 --	1.4 ± 0.38 0.8 − 2.2	1.5 ± 0.37 0.8 − 2.7
Crude protein	5.1 ± 0.70 4.1 − 6.2	4.9 ± 1.09 3.0 − 7.7	4.8 ± 0.10 4.7 − 4.9	4.9 ± 0.88 3.5 − 7.6	10.2 --	4.7 ± 0.82 3.3 − 6.6	4.9 ± 1.02 3.0 − 10.2
Crude fiber	1.2 ± 0.82 0.2 − 3.0	1.2 ± 0.61 0.2 − 2.2	0.7 ± 0.22 0.5 − 0.8	1.1 ± 0.46 0.2 − 2.4	2.5 --	1.2 ± 0.55 0.2 − 2.3	1.2 ± 0.56 0.2 − 3.0
Total Oil	11.9 ± 5.93 4.6 − 23.4	12.9 ± 5.57 5.1 − 26.5	14.9 ± 3.50 12.4 − 17.4	11.2 ± 4.85 4.3 − 27.3	3.4 --	12.7 ± 5.69 4.3 − 26.4	12.0 ± 5.32 3.4 − 27.3

X: mean value; sd: standard deviation.

In terms of oil content, Melo Neto et al. [56] found a mean value (0.93 ± 0.01%) considerably lower than that in the current study (12.0 ± 5.32%) in the scratch of *gasipaes* samples from Brazil. Arkcoll and Aguiar [23] and Alves et al. [57] determined high variability in some samples from Manaus, Brazil (2.2–61.7%) and from Acre, Brazil (7.51–24.29%), respectively. Finally, a previous study on Ecuadorian *gasipaes* fruits showed an oil content (13.28%) within the range reported in the present study [58]. Such intraspecific variation was not observed in other representative wild oleaginous palm species in the Neotropics, such as *Oenocarpus bataua* with 41.78 ± 0.39%*, Mauritia flexuosa* with 38.42 ± 1.45% [59]*, Acrocomia aculeata* with 28.94 ± 0.83% [60], and *Euterpe oleracea* with 27.7  ± 0.00% [61], but a similar variation can be observed in the oily mesocarp of *Aphandra natalia* with 31.4 ± 9.6% of oil [34]*.*

The proximate composition of fruits with different exocarp colors was generally consistent, except for the white fruits, which exhibited higher levels of moisture content (74.8%), ash (2.4%), protein (10.2%), and crude fiber (2.5%), but significantly lower oil content (3.4%) compared to fruits of other colors. However, additional samples should be included to obtain more consistent data supporting these extraordinary values.

### Intraspecific variability of phytochemical determination of mesocarp from *gasipaes* fruits

#### Total tocopherol content.

The *gasipaes* samples exhibited an average tocopherol content of 0.5 ± 0.4 mg/g (Table 2, S2 Table). These values were significantly higher than those reported by Bereau et al. [62] (0.12 mg/g) and Teixeira et al. [61] (0.059 mg/g). Moreover, the total tocopherol concentration showed considerable variation within samples of the same exocarp color, although this variability was consistent across color groups. (Table 2, S2 Table). The highest content (2.1 mg/g) was observed in a yellow-green exocarp *gasipaes* sample.

**Table 2:** Results of analysis of bioactive compounds and micronutrients in fruit mesocarp of *B. gasipaes* var. *gasipaes* (n = 110), including mean, standard deviation, and range of values.

Parameters	Mesocarp primary color						
	Green (n = 11)	Orange (n = 24)	Purple (n = 2)	Red (n = 47)	White (n = 1)	Yellow (n = 25)	All samples (n = 110)
Total tocopherol (mg/g of TO)	0.6 ± 0.40 0.1 − 1.3	0.6 ± 0.39 <LOD − 1.5	0.3 ± 0.02 --	0.5 ± 0.38 0.1 − 1.7	0.4 --	0.5 ± 0.47 0.1 − 2.1	0.5 ± 0.40 <LOD − 2.1
β-carotene (mg/100 g DW)	-- --	12.5 ± 9.90 <LOD − 27.3	<LOD --	9.4 ± 2.11 <LOD − 10.8	<LOD --	6.7 ± 3.43 <LOD − 10.5	9.8 ± 6.54 <LOD − 27.3
Sodium (mg/100 g DW)	44.6 ± 5.31 35.8 − 53.4	45.4 ± 6.05 36.2 − 61.8	43.0 ± 7.54 37.7 − 48.3	47.0 ± 8.91 31.4 − 63.8	38.0 --	45.7 ± 6.64 34.8 − 63.0	46.0 ± 7.45 31.4 − 63.8
Potassium (mg/100 g DW)	489.1 ± 97.73 334.0 − 646.0	554.9 ± 205.89 300.0 − 1132.0	432.0 ± 260.22 428.0 − 616.0	558.8 ± 125.18 350.0 − 840.0	646.0 --	531.2 ± 138.52 270.0 − 802.0	543.2 ± 148.56 248.0 − 1132.0
Calcium (mg/100 g DW)	157.1 ± 32.16 114.6 − 212.0	147.8 ± 28.08 108.4 − 220.4	154.3 ± 9.76 147.4 − 161.2	143.0 ± 21.44 103.6 − 188.6	329.4 --	144.1 ± 21.17 103.2 − 191.4	147.6 ± 29.66 103.2 − 329.4
Magnesium (mg/100 g DW)	80.7 ± 18.71 58.0 − 114.0	77.5 ± 12.47 46.4 − 100.4	63.2 ± 2.26 61.6 − 64.8	76.3 ± 9.72 61.6 − 99.8	149.4 --	76.8 ± 10.38 61.0 − 100.0	77.5 ± 13.47 46.4 − 149.4
Phosphorous (mg/100 g DW)	108.6 ± 15.57 84.0 − 132.4	117.7 ± 18.44 89.0 − 148.4	94.0 ± 3.39 91.6 − 96.4	124.6 ± 24.33 88.8 − 240.0	155.2 --	116.5 ± 18.83 82.0 − 142.8	119.4 ± 21.71 82.0 − 240.0
Sulfur (mg/100 g DW)	58.6 ± 17.02 24.5 − 78.8	57.6 ± 21.27 13.8 − 91.6	64.3 ± 15.56 53.3 − 75.3	56.6 ± 20.47 17.0 − 105.8	57.5 --	53.3 ± 17.23 22.3 − 88.8	56.4 ± 19.25 13.8 − 105.8

X: mean value; sd: standard deviation; TO: Total oil; DW: dry weight; LOD: limit of detection.

Specifically, α-tocopherol can help slow the oxidation of FAs in the exocarp, and this protective action can extend to other components, such as carotenoids, potentially altering color by preventing their degradation [63,64]. Other Neotropical palm species, such as *Oenocarpus bataua*, *Acrocomia aculeata*, *Mauritia flexuosa*, and *Aphandra nathalia*, exhibited lower tocopherol contents of 0.06, 0.2, 0.4, 0.1, and 0.45 mg/g, respectively [34,59,65,66].

#### β-carotene content.

The β-carotene values in several *gasipaes* samples were below the quantification limit (Table 2, S2 Table). However, the quantifiable samples displayed high variability, consistent with previous studies [23,67] except for Teixeira et al. [65], who reported lower values of 0.3 mg/100g in Brazilian samples. Unexpectedly, the highest *gasipaes* value (27.3 mg/100 g) corresponded to an orange-green-colored sample. While red- or orange-colored fruits have traditionally been associated with high β-carotene and tocopherol content [18], our findings showed that these samples had intermediate β-carotene levels for these colors (6.7–10.8 mg/100 g). Yellow samples ranged from 3.9 to 10.5 mg/100 g, while the purple, white, and green samples were undetectable. Indeed, previous studies have shown that red and orange fruits typically contain more bioavailable carotenoids than yellow, green, and white fruits [17,29,68]. Additionally, in different plants, the color of the fruit exocarp is associated with distinct carotenoid content [67–69]. β-carotene content of *gasipaes* far exceeded that of carrots (*Daucus carota* L.; 2.3–8.8 mg/100 g), red peppers (*Capsicum annuum* L.; 4.1–10.0 mg/kg), and melon (*Cucumis melo*; 1.6–3.6 mg/100 g) [70,71]. In addition, lower β-carotene content was found in *Oenocarpus bataua* mesocarp: 0.24 mg/100g [35], *Acrocomia aculeata:* 0.3 mg/100g [65], *Aphandra natalia:* 0.04 mg/100g [34], and *Euterpe oleracea*: 0.15 mg/100g [66].

#### Mineral content.

There is limited information regarding the mineral content of *gasipaes* mesocarp. Samples from Brazil [51,52] have reported notably lower levels of calcium, potassium, sodium, and magnesium than those in the current study; however, neither study included determinations of sulfur or phosphorus (Table 2, S2 Table). Samples from Colombia showed considerably higher mineral content than those from Brazil [50], with results similar to those of the present study, except for sodium content, which was 10 times higher in the Colombian samples. A noteworthy finding is that the white (albino) varieties exhibited a particularly high magnesium content; according to Sharma et al. [29], 100 g of cooked albino pulp can provide approximately 90% of the recommended daily intake of this mineral. Other species, such as *Oenocarpus bataua,* displayed a considerably lower mineral content, including potassium, calcium, phosphorus, and magnesium, but a higher sodium content [72]*.* Research has shown that mineral content, particularly in mesocarp tissues, varies widely among plant species. This variability is directly associated not only with the mineral content of the soil and other ecological factors [70] but also with phytochemical characteristics and genetic variations, which can impact nutrient absorption and transport. Similarly, root exudates can alter soil pH, thereby affecting mineral availability and uptake [73,74].

#### FA content.

The samples (n = 110) presented a wide range of FA content (Table 3, S2 Table). Oleic acid was the predominant FA in our analysis (43.7% ± 11.63%), followed by palmitic (30.3% ± 8.30%) and linoleic acid (15.2% ± 5.50%). Minor FA compounds included palmitoleic > α-linolenic > stearic acids. These results are inconsistent with those by Santos et al. [75] of samples from Eastern Amazonia (Marituba, Brazil), where palmitic acid was the predominant component in the mesocarp oil of red *gasipaes* fruit (50.6%), followed by oleic (35.3%) and linoleic acid (5.2%). Yuyama et al. [52] reported significant differences in mesocarp composition among three population samples of *gasipaes* from Central Amazonia, Brazil, with oleic acid being the most abundant FA in the oil, ranging from 42.8% to 60.8%. Similarly, Fernández-Piedra et al. [76] found a predominance of oleic acid with a mean of 39.2% in samples from the Germplasm Bank of the “Los Diamantes” Experimental Station from Costa Rica.

**Table 3:** Results of fatty acids profile (% of solvent-free total oil) in samples of *gasipaes* var. *gasipaes* (n = 110), including mean, standard deviation, and range of values.

Parameters (% TO)	Mesocarp primary color						
	Green (n = 11)	Orange (n = 24)	Purple (n = 2)	Red (n = 47)	White (n = 1)	Yellow (n = 25)	All samples (n = 110)
Palmitic acid (16:0)	25.5 ± 7.28 14.8 − 36.6	32.3 ± 9.69 12.4 − 49.8	20.3 ± 8.27 14.4 − 26.1	31.8 ± 6.62 16.4 − 47.5	28.2 ± 0.00 --	28.4 ± 9.11 14.0 − 46.8	30.3 ± 8.30 12.4 − 49.8
Stearic acid (18:0)	1.1 ± 0.47 0.5 − 1.9	1.8 ± 1.13 0.6 − 4.4	1.3 ± 0.66 0.8 − 1.7	1.5 ± 0.75 0.5 − 4.1	1.1 ± 0.00 --	1.7 ± 1.49 0.6 − 7.4	1.6 ± 1.04 0.5 − 7.4
**SFA**	**26.6 ± 7.62** **15.4 − 38.5**	**34.1 ± 10.53** **13.2 − 53.0**	**21.5 ± 8.93** **15.2 − 27.9**	**33.3 ± 7.06** **17.0 − 51.6**	**29.3 ± 0.00** **--**	**30.1 ± 10.01** **14.8 − 49.4**	**31.8 ± 8.95** **13.2 − 53.0**
Palmitoleic acid (16:1n7)	5.0 ± 1.46 3.4 − 8.2	6.0 ± 2.27 2.1 − 10.5	3.5 ± 0.71 2.9 − 4.0	7.1 ± 2.49 2.7 − 12.4	5.8 ± 0.00 --	5.3 ± 1.73 2.9 − 8.5	6.2 ± 2.33 2.1 − 12.4
Oleic acid (18:1n9)	47.1 ± 10.54 29.6 − 63.1	41.3 ± 13.30 3.9 − 71.5	51.7 ± 14.42 41.5 − 61.9	42.9 ± 9.51 17.1 − 67.5	20.9 ± 0.00 --	46.2 ± 13.07 22.0 − 72.1	43.7 ± 11.63 3.9 − 72.1
**MUFA**	**52.1 ± 9.63** **37.8 − 66.5**	**47.3 ± 12.31** **14.4 − 73.7**	**55.2 ± 13.70** **45.5 − 64.9**	**50.0 ± 9.20** **29.4 − 74.7**	**26.7 ± 0.00** **--**	**51.5 ± 12.97** **26.5 − 75.6**	**49.8 ± 11.09** **14.4 − 75.6**
Linoleic acid (18:2n6)	18.3 ± 5.35 9.0 − 27.0	15.8 ± 5.74 4.4 − 28.9	20.2 ± 3.37 17.8 − 22.6	13.6 ± 4.87 3.7 − 23.7	31.4 ± 0.00 --	15.2 ± 5.02 6.9 − 26.1	15.2 ± 5.50 3.7 − 31.4
α-linolenic acid (18:3n3)	3.0 ± 1.34 0.8 − 5.7	2.9 ± 1.25 0.9 − 5.6	3.1 ± 1.40 2.1 − 4.1	3.1 ± 1.55 0.7 − 7.2	12.7 ± 0.00 --	3.2 ± 2.13 0.9 − 8.8	3.1 ± 1.84 0.7 − 12.7
**PUFA**	**21.3 ± 6.62** **9.8 − 32.7**	**18.6 ± 6.66** **5.2 − 32.6**	**23.3 ± 4.77** **19.9 − 26.7**	**16.7 ± 6.00** **4.7 − 29.4**	**44.1 ± 0.00** **--**	**18.4 ± 6.50** **7.8 − 30.3**	**18.3 ± 6.83** **4.7 − 44.1**
PUFA/SFA	**0.80**	**0.55**	**1.08**	**0.50**	**1.51**	**0.61**	**0.58**
Unsaturation Index	**97.7**	**87.6**	**104.9**	**86.5**	**127.6**	**91.5**	**89.6**

X: mean value; sd: standard deviation; TO: total oil; SFA: saturated fatty acids; MUFA: monounsaturated fatty acids; PUFA: polyunsaturated fatty acids.

No differences in FA profiles were observed among the analyzed fruit samples based on exocarp color. Santos et al. [17] similarly reported that, despite the variability in fruit coloration—red, yellow, green, and white—the relative abundance of fatty acids remained consistent, with notably high levels of monounsaturated (MUFA) and polyunsaturated fatty acids (PUFA). This study corroborates those findings, showing a comparable distribution of fatty acids across all fruit color groups. Nutritional indices derived from fatty acid profiles were calculated for each fruit color, following Chen and Liu [77]. White fruits demonstrated the highest unsaturation and PUFA/SFA indices, indicating superior nutritional benefits, whereas red fruits displayed the lowest indices (Table 3). The presence of long-chain UFAs is essential for various human physiological processes, including energy production and cellular maintenance [78,79]. Consequently, consuming *gasipaes* fruits could be beneficial and may help reduce the risk of vascular and neurodegenerative diseases [79].

Other palm species with high concentrations of oleic acid on the mesocarp include *Acrocomia aculeata* (63–65% of oil), M*auritia flexuosa* (75.5% of oil), *Oenocarpus bataua* (76.7%), and *Euterpe oleracea* (63.5%). To our knowledge, such high linoleic acid levels are not typical in Neotropical palms [61]; thus, *gasipaes* fruits can be considered a good source of essential fatty acids.

#### Sterol content.

Sterol composition varied widely (Table 4, S2 Table), with sitosterol being the predominant compound. However, differences were observed in the other identified compounds. Δ5-avenaesterol was the second most abundant compound, followed by campesterol and stigmasterol. Previous research has found similar sitosterol levels in *gasipaes* fruit (exceeding 50%) [60,78,79], comparable to those observed in the present study. Oenocarpus bataua oil has been shown to contain lower levels of Δ5-avenaesterol and higher levels of campesterol [35]. The sterol content across the differently colored samples in this study remained within similar ranges, with Δ5-avenasterol and sitosterol exhibiting the most significant variability.

**Table 4:** Results of sterols (% of solvent-free total oil) analysis in samples of *gasipaes* var. *gasipaes* (n = 110), including mean, standard deviation, and range of values.

Parameters (% TO)	Mesocarp primary color						
	Green (n = 11)	Orange (n = 24)	Purple (n = 2)	Red (n = 47)	White (n = 1)	Yellow (n = 25)	All samples (n = 110)
Campesterol	9.3 ± 4.46 3.7 − 20.5	11.4 ± 3.01 6.7 − 18.0	11.3 ± 5.82 7.2 − 15.4	10.2 ± 3.18 6.3 − 21.6	10.3 ± 0.00 --	10.5 ± 3.69 5.7 − 21.5	10.4 ± 3.42 3.7 − 21.6
Stigmasterol	8.5 ± 4.43 2.7 − 14.8	9.2 ± 4.25 <LOD − 14.7	7.6 ± 7.25 2.5 − 12.8	11.1 ± 4.00 <LOD − 20.2	13.4 ± 0.00 --	11.1 ± 3.33 <LOD − 17.0	10.4 ± 4.08 <LOD − 20.2
Sitosterol	58.2 ± 6.80 47.0 − 73.1	52.4 ± 5.24 43.2 − 61.9	46.0 ± 6.48 41.4 − 50.6	53.4 ± 5.62 39.6 − 73.7	46.0 ± 0.00 --	52.7 ± 6.76 40.1 − 74.6	53.3 ± 6.16 39.6 − 74.6
Δ5-Avenasterol	22.1 ± 6.25 9.9 − 27.6	22.2 ± 5.25 9.1 − 33.2	29.0 ± 9.23 22.5 − 35.5	21.4 ± 7.32 7.5 − 36.9	25.9 ± 0.00 --	22.2 ± 7.32 8.4 − 36.1	22.0 ± 6.76 7.5 − 36.9

X: mean value; sd: standard deviation; LOD: limit of detection.

Variability in plant sterol content is high among the fruits of different mesocarp colors and is directly influenced by environmental and developmental signals [80,81]. Plant sterols have garnered increasing interest in the food industry given their presence in many staple vegetables and their potential benefits for human health, including anti-aging, anticancer, antioxidant, and antiatherogenic effects [82]. This nutritional potential (content of amino acids, fiber, vitamins, minerals, FA, and sterols) suggests its capacity of *gasipaes* fruits to be used for food and nutraceutical purposes.

#### Correlations between the phenotypic characteristics and the phytochemical and proximal composition.

Nonparametric Spearman’s correlation analysis revealed several significant, albeit low (S4 Table), correlations between various phenotypic characteristics and the composition of gasipaes mesocarp samples (n = 110).

First, regarding nutritional composition, the mean fruit size (in terms of weight and volume) was inversely correlated with tocopherol content (r = −0.333, p = 0.002, and r = −0.357, p = 0.001, respectively). Fruit moisture content (n = 110) was directly correlated to crude protein (r = 0.511, p = 0.000), crude fiber (r = 0.328, p = 0.000), K (r = 0.570, p = 0.000), Ca (r = 0.248, p = 0.009), P (r = 0.410, p = 0.000), and Mg content (r = 0.392, p = 0.000), highlighting the importance of water availability for nutrient uptake in palm fruit.

Fruit total oil content was inversely correlated with tocopherol (r = −0.461, p = 0.000) and PUFA content (r = −0.680, p = 0.000) but directly correlated with MUFA content (r = 0.534, p = 0.000), which is inconsistent with [43,83], who found an association between oil content and PUFA. Finally, the analysis revealed a noteworthy direct association between PUFA (primarily α-linolenic acid) and tocopherol content (r = 0.434, p = 0.000), highlighting the nutritional benefits of incorporating these nutrients into the daily diet.

Previous correlation studies are scarce and generally limited to fruit size, texture, and mesocarp oil content. The mesocarp of small fruits tends to be more fibrous and oilier, while that of large fruits tends to be rich in starch and lower in oil [50,53,75,83].

#### Significant differences among orange, red, yellow, and green gasipaes samples.

The Kruskal–Wallis test was applied to assess differences in phytochemical and nutritional parameters among samples grouped by exocarp color. In most cases, the test revealed no statistically significant differences between the color categories. The significance levels for components such as water, ash, protein, fiber, total oil, tocopherols, carotenoids, and minerals (including potassium, calcium, phosphorus, magnesium, sulfur, and sodium) were above the 0.05 threshold, thereby retaining the null hypothesis in each case. These results support retaining the null hypothesis and suggest a relatively uniform distribution of these compounds across fruit colors. However, a significant exception was observed for palmitoleic acid (p = 0.004), in which the null hypothesis was rejected, suggesting that this fatty acid exhibits significant variation across color groups. This specific finding underscores the importance of considering specific compounds when evaluating the relationship between fruit color and chemical composition.

However, as highlighted in the results, β-carotene presents a distinct scenario. Although statistical comparisons across all color categories were not feasible due to their absence in several groups, the presence and accumulation of β-carotene constitute a clear phenotypic difference. Among the quantifiable samples, variability was pronounced: the highest concentration was unexpectedly observed in an orange-green fruit, whereas orange-red fruits exhibited intermediate levels, yellow fruits showed lower concentrations, and purple and white fruits were below detection limits. These findings demonstrate that exocarp color alone does not reliably predict β-carotene content, underscoring the complexity of pigment biosynthesis and accumulation in *Bactris gasipaes*.

Taken together, the absence of broad biochemical differentiation among color categories indicates that exocarp color is not the primary driver of this diversity. In addition to color, many other genetic and epigenetic factors may contribute to the variability observed in these samples. Ecological influences may also play a role; nevertheless, the study area is relatively homogeneous in terms of climatic and edaphic conditions, with altitude representing the most pronounced environmental gradient (between 500–1110 masl). Populations were distributed between 200–400 m (n = 60) and 600–1000 m (n = 42), with only a few samples (n = 10) from intermediate or higher elevations (>1000 m). This altitudinal variation, although limited, may influence certain traits and warrants further investigation. Additionally, the PCA (S5 Fig) showed no association pattern among samples, suggesting that spatial variation has not influenced structuring local diversity in gasipaes samples. A deeper understanding of these drivers—particularly the genetic and epigenetic processes underlying phenotypic variation—will require population-genetic approaches beyond the scope of the present study.

## Conclusions

*Bactris gasipaes,* particularly its mesocarp, exhibits remarkable phenotypic and phytochemical diversity, being rich in unsaturated fatty acids, fiber, tocopherols, and total carotenoids. Although its protein content is modest, the presence of all essential amino acids enhances its nutritional value. Nevertheless, correlating the content of any particular biomolecule with the final coloration of the fruit exocarp is challenging because of the multitude of compounds that contribute to the final pigmentation. Comprehensive chemical characterization of fruits with different exocarp colors is essential for promoting their use in food, oil, flour, and dietary supplements. Such data are also critical for the selection, conservation, and sustainable use of genetic resources and for the enforcement of food systems. While diversity offers ecological and cultural advantages, excessive variability can pose challenges for industrial standardization, underscoring the need for deeper insights into the biochemical and biophysical factors shaping fruit composition.

The urgency to conserve *B. gasipaes* diversity in Ecuador is growing, as commercial monocultures, climate change, and the erosion of traditional knowledge threaten its genetic variability. Regional initiatives should prioritize documenting this diversity and developing food applications that respect agroforestry systems and local communities. In areas with limited access to nutritious oils, *B. gasipaes* emerges as a promising alternative to improve food security and health. Furthermore, comprehensive chemical characterization of fruits with different exocarp colors is essential to promote their use in food, oil, flour, and dietary supplements. Such data are critical for the selection, conservation, and sustainable utilization of genetic resources, as well as for understanding the biochemical and biophysical factors shaping fruit composition.

Beyond its nutritional and cultural importance in the local food systems, *B. gasipaes* serves as a cultural keystone species in Amazonian landscapes. Its integration into agroforestry systems supports biodiversity and traditional ecological knowledge while strengthening community resilience in regions increasingly affected by illicit economies. Recognizing its biocultural legacy is crucial for guiding future research, conservation strategies, and development policies.

## Supporting information

S1 Appendix Summary of proximal and chemical analyses performed on *Bactris gasipaes* var. *gasipaes* mesocarp samples. (DOCX)

S2 Table Information and results of determinations from *Bactris gasipaes* var. *gasipaes* samples. (XLSX)

S3 Fig Dark purple sample of *Bactris gasipaes* var. *gasipaes* collected in Jondachi locality. (PNG)

S4 Table Results of Spearman's non-parametric correlations, significance level and number of data points in the comparison between the phenotypic and phytochemical variables of the fruits of Bactris gasipaes var. gasipaes. (XLSX)

S5 Fig Principal Component Analysis (PCA) of fruit traits in *Bactris gasipaes* var. *gasipaes*: PC1 vs. PC2 scatterplots colored by exocarp first color. (PNG)
