# Supplementary material for: High phenotypic and phytochemical diversity of Bactris gasipaes (Arecaceae) fruits in Ecuador
Source: PLoS One. 2026 Mar 26;21(3):e0342904. doi: 10.1371/journal.pone.0342904 (PMC13020821; doi:10.1371/journal.pone.0342904)
Supplement: S1 Appendix — (DOCX) [file pone.0342904.s001.docx]

S1 Appendix: Summary of proximal and chemical analyses performed on *Bactris gasipaes* var. *gasipaes* mesocarp samples

| **Parameter** | **Method** | **Conditions** | **Calibration curves** | **Reference** | **Units** |
| --- | --- | --- | --- | --- | --- |
| Ashes  (mineral content) | Gravimetric | Calcination at 550 °C for 3 hours | Not applicable | ISO 5984 (2022) | % DW |
| Crude protein | Kjeldahl method | Approximately 1 g of the sample underwent digestion (JP Selecta Block, Barcelona, Spain) with sulfuric acid, potassium persulfate, and mercuric oxide as catalysts (all analytical grade).  Nitrogen steam distillation (JP Selecta Pro-Nitro Distiller) was collected in a 40 % sodium hydroxide solution, analytical grade.  Finally, a standardized hydrochloric acid solution was used for the titration.  The conversion factor used for protein calculation was 6.25. | Not applicable | ISO 5983-1 (2019) | % DW |
| Crude fiber | Gravimetric | Degreased samples (dried and milled) underwent acid hydrolysis, washing, and subsequent basic hydrolysis using a cellulose and fiber determination extractor (JP Selecta 4000623, Barcelona, Spain). The dry residues were then calcinated at 550 °C for 3 hours. | Not applicable | ISO 6865 (2022) | %DW |
| Total lipids | Folch method | Solid-liquid extraction and gravimetric determination of the dried total oil (TO). | Not applicable | Romero‐Estévez et al. 2023 | %DW |
| Total tocopherols | Direct high-performance liquid chromatography (HPLC) | Ten milligrams of TO was dissolved in 1000 µL of n-hexane and directly injected into the HPLC system (LaChrom Elite, Hitachi, Japan) with a fluorescence detector using:  A Merck Lichrospher Si-60 (250 × 4 mm internal diameter, 5 µm particle size) column.  Mobile phase: n-hexane / propane-2-ol (99:1, v/v).  Injection volume: 30 µL  Flow rate: 1 mL/min.  Excitation wavelength: 290 nm.  Emission wavelength: 330 nm.  Calibration with commercial standards (Sigma Aldrich). | Equation: y = ax + b  a = 3.60769e-0.008  b = -0.0581905  r^2^ = 0.998985 | Modified method from Montúfar et al. (2010). | mg/g TO |
| Fatty acids (FA) profile | FAs were derivatized and analyzed as methyl esters (FAME) using gas chromatography with a flame ionization detector (GC-FID) | The hexane phase containing FAMEs was filtered through a PTFE 0.5 µm syringe filter, and injected into a gas chromatograph (GC; Clarus 500, PerkinElmer, Shelton, CT, USA) equipped with flame ionization detection (FID) using:  Famewax capillary column (Restek, Lisses, France, 30 m × 0.25 mm × 0.25 mm).  Heating program:  100 °C for 2 min,  140 °C at a rate of 10 °C/min,  190 °C at a rate of 3 °C/min,  260 °C at a rate of 30 °C/min, which was maintained for 2 min.  Carrier: Helium at a flow rate of 40 cm/s.  Injector temperature: 230 °C.  Detector temperature: 260 °C.  FAME identification was performed using a commercial mixture standard (37-component FAME Mix C_4_-C_24,_ Supelco) | Results were calculated as the individual peak area of ​​each component over the total area of ​​the peaks. | Laffargue et al. (2007) | % TO |
| Sterols profiles | Saponification, thin-layer chromatography (TLC) and gas chromatography with flame ionization detector (GC-FID) | Saponification with 2 mol/L ethanolic (5%) sodium hydroxide heated at 90°C for 60 minutes.  Liquid-liquid separation with diethyl ether three times, and the upper phase was dried under a nitrogen flow (Glas-Col, Terre Haute, IN, USA) until constant weight.  Thin-layer chromatography using TLC plates (silica 60G, F_254_, 20 x 20 cm) using:  A solvent system of n-hexane/diethyl ether (1/1, v/v).  A 0.1% 2,7-dichlorofluorescein ethanolic solution as a developer.  Identification using UV light (366 nm) by comparing the retention factors using cholesterol standard as an internal standard.  The identified band was scraped off the plate and transferred to 4 mL of diethyl ether.  Sterol profiles were analyzed using the FID-GC:  A SAC-5 capillary column (Supelco, France, 30 m x 0.25 mm x 0.25 μm) at 300 °C.  Carrier: Helium at a flow rate of 1.2 mL/min.  Both injector and detector temperatures were set at 285 °C.  Sterols were identified by comparing them to commercial standards (Sigma Aldrich). | Results were calculated as the individual peak area of ​​each component over the total area of ​​the peaks. | Modified method from Montúfar et al. (2010). | % TO |
| β-carotene  [analyzed in a subset of gasipaes samples n=20, representing the primary colors collected in the study area (i.e., red, orange, yellow, green, white, and purple)]. | Liquid-liquid extraction and HPLC with a Diode array detector (DAD) | 0.5 g of lyophilized sample was mixed with 10 mL of acetone, underwent ultrasonic treatment using an ultrasonic bath (40 kHz, 30 min, ±2 °C, 22FS30D, Fisher Scientific, Hampton, NH, USA), and centrifuged for 15 min (10,000 min^-1^, Sorvall ST 8, Thermo Fisher Scientific, Asheville, NC, USA) to separate the supernatant. Then the supernatants were filtered using regenerated cellulose syringe filters (0.2 μm, Millipore, Bedford, MA, USA). And injected to HPLC system (Dionex UltiMate 3000, Thermo Fisher Scientific, Waltham, MA, USA):  SunFireTM C18 column (3.0 × 150 mm, 5 μm)  Mobile phase: methanol and acetonitrile served as solvents A and B with a flow rate of 1mL/min.  Injection volume 10 μL.  A multi-step linear gradient was employed:  0.0 min [A: B 50/50],  6.0 min [A: B 0/100],  9.0 min [A: B 0/100],  and 13.0 min [A: B 50/50].  Column temperature: 25 °C.  Autosampler temperature 4 °C.  Detector: 457 nm using a photodiode array detector scanning from 200 to 600 nm, with a resolution of 1.2 nm.  Calibration using a commercial standard. | Equation: y = a x + b  a = 0.097  b = 0.084  r^2^ = 0.995 | Carvalho et al. (2013) | mg/100 g DW |
| Minerals: calcium (Ca), magnesium (Mg), potassium (K), and sodium (Na) | Acid-wet digestion and Flame atomic absorption spectroscopy (FAS) | Dried samples were prepared using an acid-wet digestion process with analytical grade nitric acid.  External calibration curves for all elements were generated using AccuStandard® standards. | Equation: y = a x + b  Calcium:  a = 0.0109  b = 0.0075  r^2^ = 0.999  Magnesium:  a = 0.0972  b = 0.0085  r^2^ = 1.000  Potassium:  a = 0.03118  b = 0.0105  r^2^ = 0.999  Sodium:  a= 0.0758  b= 0.006  r2= 0.999 | AOAC 975.03 (1988) | mg/100 g DW |
| Phosphorus (P) | Molybdovanadate phosphate method | The digested samples were prepared from the AOAC 975.03 method using a UV-Vis spectrophotometer (V-630, Jasco, Tokyo, Japan).  External calibration curves for all elements were generated using AccuStandard® standards. | Equation: y = a x + b  a = 0.067  b = -5.55112e-017  r^2^ = 1.000 | AOAC 958.01 1958 | mg/100 g DW |
| Sulfur (S) | Turbidimetric analysis | Direct turbidimetric analysis was performed using the UV-Vis spectrophotometer, using the mineral solution obtained from the digested samples prepared as per AOAC 975.03.  External calibration curves for all elements were generated using AccuStandard® standards. | Equation: y = a x + b  a = 0.0766  b = 0.015  r^2^ = 0.997 | Tabatabai and Bremner (1970) | mg/100 g DW |
