# Supplementary figures and images for: High phenotypic and phytochemical diversity of Bactris gasipaes (Arecaceae) fruits in Ecuador
Source: PLoS One. 2026 Mar 26;21(3):e0342904. doi: 10.1371/journal.pone.0342904 (PMC13020821; doi:10.1371/journal.pone.0342904)

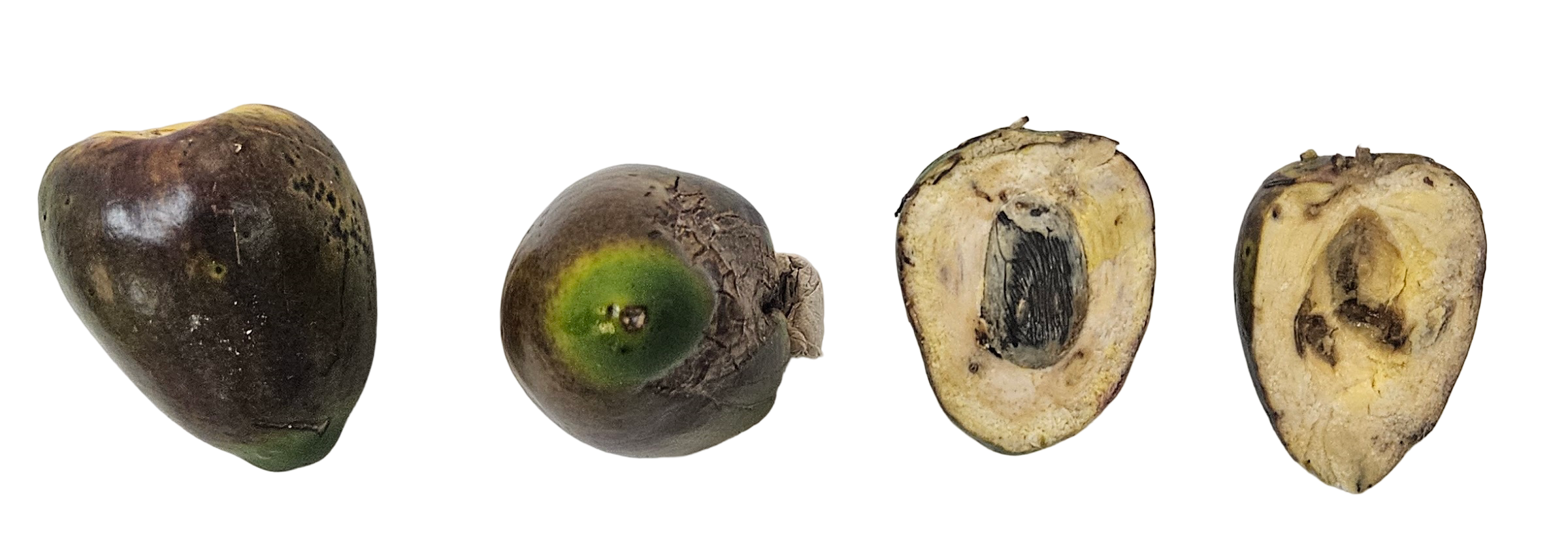

Supplement: S3 Fig — (PNG) [file pone.0342904.s003.png]

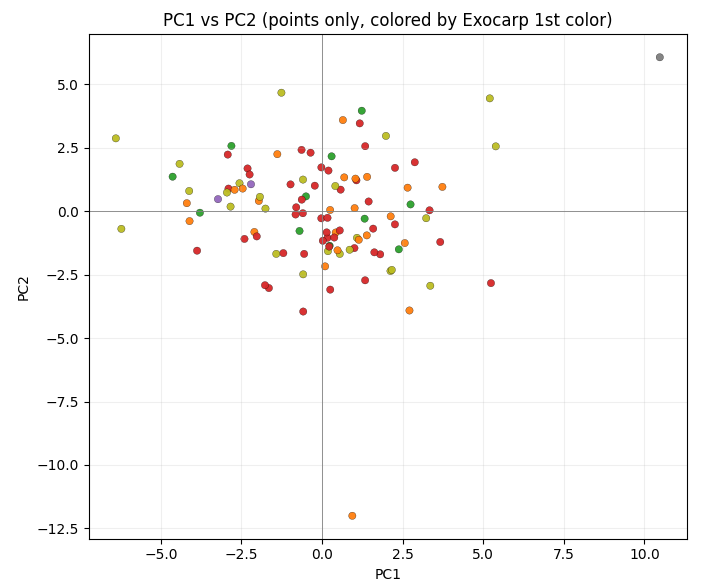

Supplement: S5 Fig — (PNG) [file pone.0342904.s005.png]
